# Metabarcoding–approach–based profiling reveals dynamic nature of sustainable tillage practices on nematode communities in corn–soybean cropping systems

**DOI:** 10.1038/s41598-025-09356-6

**Published:** 2025-07-14

**Authors:** Jerry Akanwari, Md Rashedul Islam, Ping Liang, Tahera Sultana

**Affiliations:** 1 Department of Biological Sciences, Brock University, St. Catharines, ON L2S 3A1 Canada; 2 London Research and Development Centre, Agriculture and Agri–Food Canada, Vineland Station, ON L0R 2E0 Canada; 3 National Microbiology Laboratory, Public Health Agency of Canada, Winnipeg, MB R3E 3R2 Canada

**Keywords:** Tillage, Corn–soybean, Nematode community profiling, Ecological indicator, Soil health, Ecology, Plant sciences, Environmental sciences

## Abstract

Despite extensive research on tillage and its impact on nematode communities, little is known about the impact of occasional tillage within no–tillage practices. Occasionally tilling the soil can minimize the negative effects of conventional tillage and no–tillage practices especially in clayey soils. A tillage system that maximizes all the economic and environmental benefits but increases herbivore nematodes while reducing beneficial free–living nematodes may pose long–term agronomic challenges. In this study, we investigated the impact of conventional tillage (CT), minimum tillage (MT), and occasional tillage within no–tillage (NT) systems, on nematode communities in corn–soybean cropping systems in Ontario, Canada using metabarcoding approach. Soil samples were collected at 0–5 cm and 5–20 cm depths during planting and before harvesting of corn/soybeans in 2021 and 2022. The results showed that tillage significantly influenced nematode community structure and distribution within the soil profile. Beneficial free–living nematodes were abundant at 0–5 cm (> 70%), while herbivores dominated at 5–20 cm. The MT and NT systems supported a higher relative abundance of bacterivores, particularly *Rhabditis*, at both depths. Bacterivore populations were 1.7 times higher in MT than in CT at the corn/soybean maturity stage. The CT system favoured herbivore nematodes, especially *Pratylenchus*, with 47% higher populations at 0–5 cm and 76% higher at 5–20 cm compared to MT and NT. The CT was initially characterized by high maturity index and structure index values, possibly due to legacy effects; however, both metrics declined over time, whereas values under NT increased. The plant–parasitic index was elevated in both CT and NT but differed in the dominant functional guilds. The nematode channel ratio was higher under CT and MT, signaling a stronger bacterial pathway, while NT gradually shifted toward a more fungal microbial channel. The soil organic matter (OM), total carbon and total organic carbon were significantly higher at the 0–5 cm depth in the MT and NT systems. Correlation analysis identified pH, OM, total nitrogen and cation exchange capacity as the key soil properties shaping the structure of nematode communities. Our findings suggest that MT and NT can enhance soil health and long–term resilience against herbivores in corn–soybean cropping systems.

**Supplementary Information:**

The online version contains supplementary material available at 10.1038/s41598-025-09356-6.

## Introduction

 Nematodes are widely distributed multicellular organisms that significantly influence ecosystem processes at various trophic levels. Changes in their community composition can affect essential ecological functions, including nutrient cycling, plant nutrient uptake, organic matter decomposition, and the regulation of diseases and pests^1–3^. Nematode communities in soils of agricultural ecosystems have already been investigated, especially as indicators of ecosystem structure and function^3,4^. They are widely employed to track the ecological effects of different agricultural and environmental management practices on soil health conditions^5^.

Nematodes, being bacterivores, fungivores, omnivores and carnivores serve as one of the most cost-efficient and effective biological indicator tools. This is due to their sensitivity to environmental changes, even within the same feeding groups, as well as other unique biological characteristics^6^. As a result, nematodes have been used to advance our understanding of ecosystem responses to environmental pollution and agricultural sustainability programs^4,7,8^. The use of nematodes as ecological indicators is measured by applying indices such as the maturity index (MI), structure index (SI), enrichment index (EI), channel index (CI), and nematode channel ratio (NCR) to analyze soil health. Although the SI examines soil stability and its capacity to moderate herbivores, fungivores, and bacterivores, the MI measures the extent of soil disturbance of the soil. The CI and NCR distinguish between bacteria– or fungi–dominated breakdown pathways, whereas the EI indicates resource availability^9–11^. The indices are used extensively to quantify the services and functions of soil ecosystems^11–13^.

Increasing interest in farmland productivity and environmental conditions highlights the importance of sustainable management^14^. Globally, conservation tillage and no–tillage are increasingly being adopted in place of conventional tillage (CT)^15^. Conservation tillage and no–tillage practices decrease the runoff of nutrients, and enhance environmental sustainability through increased soil organic matter, greenhouse gas reductions, and increased biodiversity^16–18^. More than 80% of Canadian cropland is farmed using conservation tillage and no–tillage, with no–tillage accounting for over 60% ^15^. In Ontario, the use of no–tillage is increasing with over 66% of farmland utilizing conservation tillage and no–tillage systems^15,19^. In spite of the economic and environmental benefits of no–tillage, its adoption in clayey soils is often challenging due to compaction and weed management^20–22^. To mitigate these challenges, occasional tillage within the no–tillage system (NT) is recommended^22,23^. The NT system can help growers integrate other management practices such as cover crops and manure, which are commonly used in many countries^4,24,25^. Many corn–soybean farmlands in Ontario are characterized by high clay content that requires maximizing tillage strategies for better soil health and agricultural sustainability. This is determined by our ability to understand the influence of different practices on belowground communities, which are essential for ecosystem functioning.

Although there are several studies on the effects of conservation tillage and no–tillage on nematode community structure^21,25,26^, little is known about the impact of the NT system. Therefore, the objective of this study was to investigate the variation in nematode population diversity at various soil depths under different tillage management systems in a clayey soil in corn–soybean rotation. We examined three tillage systems, where the plots were managed conventionally with tillage (CT), with minimum tillage (MT), and continuous no–tillage with occasional tillage (NT), a strategic approach commonly used in clay-rich soils. The current research hypothesized - (i) bacterivore nematodes would be most abundant in the CT system due to crop residue incorporation, and (ii) predator nematodes would be abundant in the NT system due to the minimal disturbance.

## Materials and methods

### Site description

The research was conducted in Wallenstein, Ontario, Canada (43° 38’ 59.568’’ N, 80° 37’ 14.628’’ W) at a long–term corn–soybean rotation site that incorporates a clover mix/rye/wheat/barley as winter cover crops. The site had been managed using CT for over 10 years. All soybean varieties used at this site possessed resistance to soybean cyst nematode. The soil was classified as Brookston clay loam, with an average composition of 26% sand, 36% silt, and 38% clay.

## Experimental design and soil sampling

**Fig. 1:**
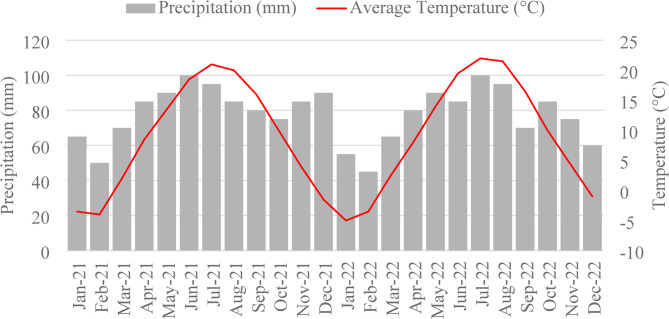
Monthly average temperature (^o^C) and precipitation (mm) for 2021 and 2022 crop season.

The experiment was arranged in a randomized complete block design, with tillage–based management systems as the primary treatment factor. The experimental plots were 10 m wide × 35 m long, and each treatment was replicated four times. The treatments were − (1) Conventional tillage (CT): tillage using a Mouldboard plough, Kongskilde cultivator, followed by a high–speed disc pass; (2) Minimum tillage (MT): annual tillage using a Kongskilde cultivator and high–speed disc pass; and (3) Occasional tillage within no–tillage (NT), plots were managed under no–tillage but received a high–speed disc pass in 2019 and 2022. The three different tillage treatments were implemented in 2017 within a corn–soybean rotation. In the CT and MT treatments, tillage was carried out prior to the planting of winter cover crops and the main crop. The NT treatment had no winter cover crops. Liquid manure was surface applied annually as part of standard tillage planting and agronomic practices across all treatments before planting of the main crop. Weed management was occasionally carried out using herbicides. Pesticides were applied as needed to control pest populations. Field management activities for each system are provided in Supplementary Table S1. The temperature and precipitation data from January 2021 to December 2022 are shown in Fig. 1.

Experimental plots were sampled during planting and before harvesting (April and September, respectively) in 2021 and 2022. Soybean was the main crop in 2021, and corn was planted in 2022. A standard 2.5 cm diameter soil probe was used to randomly collect 10 soil cores from each plot at a depth of 20 cm. The soil cores were divided into 0–5 cm and 5–20 cm depths, and each depth was pooled across the ten cores to create a composite sample. Extra soil samples were taken for nutrient analysis. All soil samples were placed in labeled polyethylene sample bags and transported to the laboratory on dry ice. The samples were kept at 4 ^o^C until processing. A total of 96 soil samples were collected (3 Treatment x 4 replications (block) x 2 depths x 2 sampling times x 2 years) for this study.

## Soil nutrients analysis

Soil samples collected for nutrient analysis were sent to A&L Canada Laboratories Inc. (London, Ontario, Canada) for the determination of soil pH, organic matter (OM), cation exchange capacity (CEC), total carbon (TC), total organic carbon (TOC), and total nitrogen (TN). All analyses and their respective methods are listed in Supplementary Table S2.

## Nematode isolation from soil

Soil samples were gently homogenized by sieving through a 5 mm mesh to remove coarse materials such as stones and plant debris. Between samples, sieves were rinsed under hot tap water to clean them. 50 g of each replicate was used to extract nematodes using the centrifugation and sugar flotation methods^29^. Prior to DNA extraction, extracted nematodes were kept at − 20 °C after being submerged in liquid nitrogen for five seconds^6^.

## DNA extraction and amplicon sequencing

Genomic DNA was extracted from the isolated nematodes using the DNeasy Blood & Tissue Kit (Cat: 69504, Qiagen Inc., Mississauga, ON, Canada) following the manufacturer’s instructions, except that after adding the 180 µl of Buffer ATL and 20 µl Proteinase K, the mixture was vortex and placed in a 55 °C incubator overnight. The concentration of DNA was quantified using a Nano spectrophotometer (Model: DS–11 FX FroggaBio, Concord, ON, Canada). An average of 20 ng/µL DNA was sent to Génome Québec (Génome Québec, Montréal, Québec, Canada) for PCR amplification, library preparation and sequencing using NF1 (GGTGGTGCATGGCCGTTCTTAGTT) and 18Sr2b (TACAAAGGGCAGGGACGTAAT) primer pairs^30^. Paired–end sequencing was performed on an Illumina MiSeq platform to generate 2 × 300 bp paired–end reads. The primer pair, which covers the V6–V8 region of the 18 S rRNA gene, is one of the most widely used primer pairs for nematodes metabarcoding studies^21,30^.

### Bioinformatics analysis

After receiving the raw sequence data from the sequencing service provider, they were uploaded to the graham cluster of Digital Research Alliance of Canada. The fastp plugin was used to remove primers and low–quality reads^31^. Forward and reverse reads were trimmed exactly at 280 and 270 bp, respectively, with a maximum *N* = 0 and maximum EE = 2. The nf-core/ampliseq Nextflow pipeline (version 2.7.1) was utilized to process the data^32,33^. A 10 bp overlapped minimum read length was used for the end–to–end merging of the trimmed reads. Subsequently, a Naive Bayes q2–feature–classifier was employed to assign taxonomy to the OTUs^34^. The classifier was trained on the NemaTaxa database using sequences clustered at 99% species identity threshold^35^. Most ecological studies on nematode metabarcoding support the use of 99% similarity benchmark^21,36^.

## Nematode alpha diversity, trophic groups and ecological indices

Alpha–diversity indices, including Richness, Shannon, and Simpson, were calculated using the “microeco” package in R (v1.11.0)^37^. Observed OTUs abundance data were uploaded to the Nematode Indicator Joint Analysis (NINJA) web program (https://shiny.wur.nl/ninja/, accessed on September 21, 2024)^38^. The NINJA software was then used to classify trophic groups, such as herbivores, fungivores, bacterivores, and predators (carnivores and omnivores). It was also employed to compute nematode ecological indices, such as the MI, EI, SI, CI, and plant–parasitic index (PPI). The NCR was calculated using the formula $\:NCR=\frac{Ba}{Ba+Fu}$ where Ba represents the abundance of bacterivore and Fu represents the abundance of fungivore nematodes.

### Statistical analysis

All statistical analyses were performed with R software version 4.3.1^39^. All Figures were generated using the ‘ggplot2’ version 3.5.1^40^. Data analysis was conducted using non–rarefied OTUs, with normalization based on relative abundances to account for differences in sequencing depth. The relative abundance for each taxon was calculated by dividing the number of OTUs assigned to a taxon by the total number of OTUs in each sample. The effect of tillage on nematode communities was analyzed using mixed–effects models, where tillage type, sampling depth, time of sampling, and the interaction between tillage type and sampling time were fitted as fixed effects. The ‘lme’ function from the ‘nlme’ package was used, with a nested random effect structure defined as block within year^41^. Starting with a null model that simply included the random effect, forward selection was used to find the most parsimonious model. The model with the lowest Akaike information criterion (AIC) was selected after fixed effects were introduced one after the other.

To test for tillage type variation, sampling depth, and sampling time, analysis of variance (ANOVA) was utilized. Levene’s test was utilized to confirm variance homogeneity, and Q–Q plots were used to confirm residual normality. Data were square root– or log(x + 1) transformed as necessary to satisfy model assumptions. For significant ANOVA findings (*P* < 0.05), post–hoc tests were carried out using the Tukey–Kramer test^42^. Applying the ‘fortify_mantel’ function of the ‘ggcor’ and’vegan’ packages, a Partial Mantel test was performed to investigate the relationship between soil properties and nematode feeding groups^43^. Non-metric multidimensional scaling (NMDS) using Bray-Curtis dissimilarities was used to determine the effect of tillage on nematode communities^44^. The analysis was conducted using the *adonis2()* function in the vegan package with 999 permutations^45^. The effect of tillage, sampling depth, and soil properties on the composition of nematode communities was examined through redundancy analysis (RDA).

## Results

### Effects of tillage on nematode communities

**Fig. 2:**
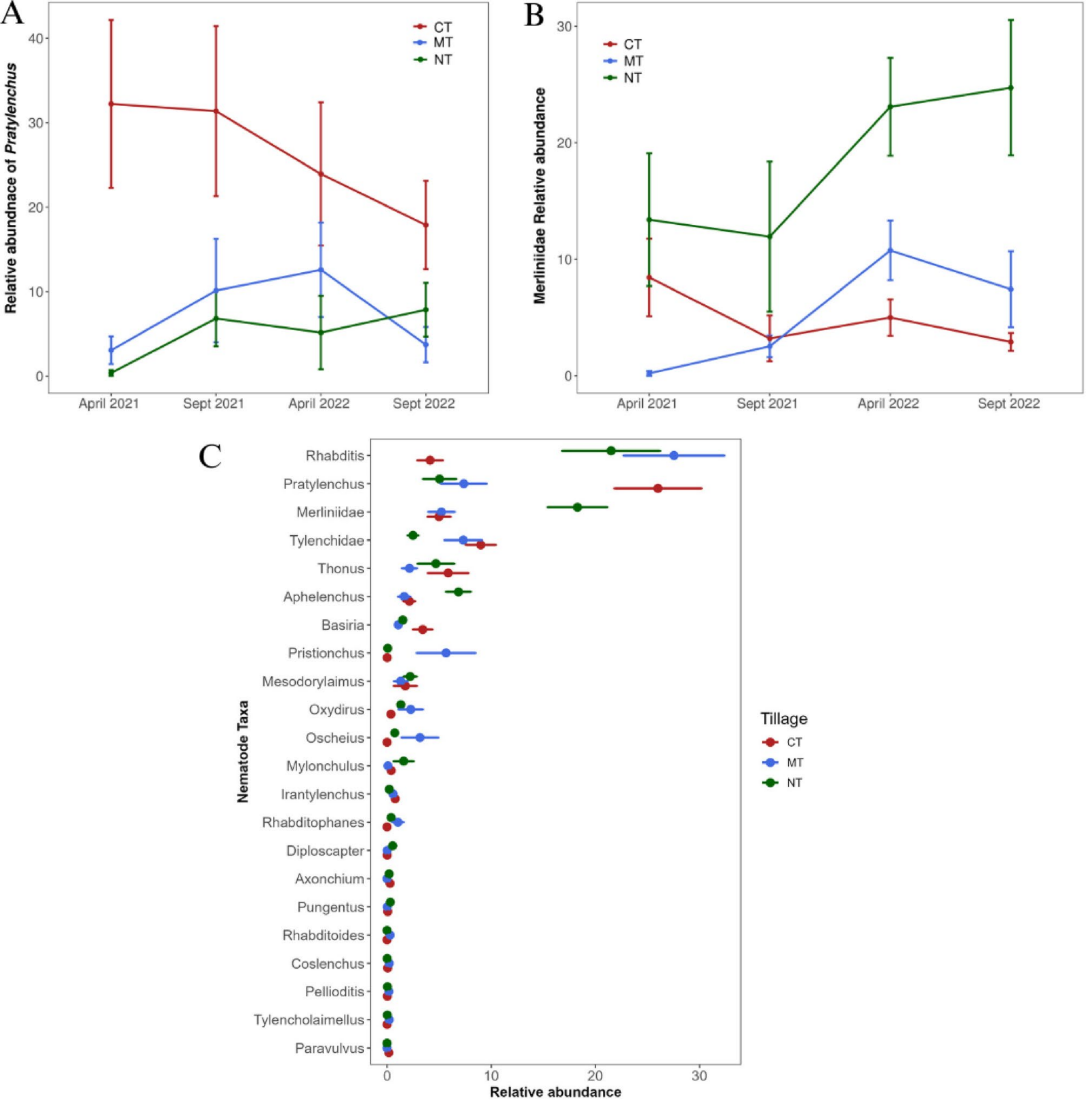
Variation in the relative abundance of *Pratylenchus* spp., (**A**) and Merliniidae spp., (**B**) and species abundance comparison (**C**) across sampling times. Individual point represents average (± standard error) soil nematodes for each sampling time. Significance of C was tested using Kruskal Wallis test (*P* < 0.05) and method of p.adjustment = “BH”.

The current research identified 57 nematode taxa, all of which were present at the 0–5 cm soil depth, while 51 of them were also present at the 5–20 cm depth. The relative abundance of nematode genera was significantly affected both by tillage type and sampling depth (Table 1). Free–living nematodes were generally abundant at the 0–5 cm depth e.g.,* Eucephalobus* was significantly abundant at 0–5 cm across all tillage types (*P* < 0.001). Similarly, *Alaimus* exhibited significantly higher relative abundance under CT, while *Aphelenchus* was prevalent at the 0–5 cm depth across all tillage systems. *Boleodorus* was abundant in MT at the 5–20 cm depth, whereas *Pratylenchus* and *Basiria* were more abundant in the CT system at both depths. *Pratylenchus* was the most enriched herbivore taxon, and although CT favoured its proliferation by 66%, there was a consistent decline in its population over the sampling period (Fig. 2A). The relative abundance of *Merliniidae* spp. was significantly higher in the NT treatment and had an average increase > 1.8 fold across the sampling time (Fig. 2B). *Oscheius* and *Pristionchus* were significantly abundant at 0–5 cm (*P* < 0.05) under the MT management system. Additionally, *Rhabditis* had the highest relative abundance in MT and NT systems at the 0–5 cm depth (Table 1; Fig. 2C).

**Table 1:** The mean relative abundance (%) of nematode genera associated with different tillage systems and depth of soil sampling.

	0–5 cm			5–20 cm		
	CT	MT	NT	CT	MT	NT
Bacterivores						
*Acrobeloides*	1.97 ± 0.73	0.91 ± 0.25	1.89 ± 0.37	0.84 ± 0.20	0.76 ± 0.25	1.36 ± 0.45
*Alaimus*	2.76 ± 1.19a	0.55 ± 0.19b	0.57 ± 0.25b	0.25 ± 0.15b	0.19 ± 0.16b	0.15 ± 0.06b
*Eucephalobus*	12.72 ± 2.97a	11.72 ± 2.29ab	7.62 ± 1.28bc	3.21 ± 1.62 cd	2.17 ± 0.63d	1.70 ± 0.41d
*Eumonhystera*	0.05 ± 0.03	0.01 ± 0.01	0.21 ± 0.14	0.62 ± 0.58	0.03 ± 0.02	0.18 ± 0.09
*Oscheius*	0.01 ± 0.01b	5.71 ± 3.41a	1.31 ± 0.38b	0.00 ± 0.00b	0.61 ± 0.38b	0.17 ± 0.13b
*Panagrolaimus*	0.75 ± 0.68	0.10 ± 0.07	0.40 ± 0.19	0.02 ± 0.0	0.00 ± 0.0	0.10 ± 0.06
*Plectus*	0.76 ± 0.22b	2.13 ± 0.75ab	0.70 ± 0.23b	1.71 ± 0.46ab	2.78 ± 1.06ab	3.59 ± 1.16a
*Rhabditis*	2.70 ± 1.69b	25.47 ± 6.67a	28.65 ± 7.20ba	5.39 ± 1.71b	29.64 ± 7.15a	18.39 ± 6.14ba
*Rhabditophanes*	0.00 ± 0.00	1.49 ± 1.01	0.27 ± 0.21	0.00 ± 0.00	0.61 ± 0.31	0.52 ± 0.42
*Diploscapter*	0.02 ± 0.02	0.05 ± 0.03	0.26 ± 0.12	0.02 ± 0.02	0.00 ± 0.00	0.82 ± 0.66
**Fungivores**						
*Aphelenchoides*	0.43 ± 0.11ab	1.26 ± 0.61a	0.19 ± 0.05b	0.50 ± 0.25ab	0.42 ± 0.15ab	0.16 ± 0.05b
*Aphelenchus*	2.76 ± 0.81b	1.09 ± 0.47b	9.20 ± 1.92a	1.59 ± 0.75b	2.20 ± 1.04b	4.50 ± 1.17cb
*Ditylenchus*	5.18 ± 1.51a	1.66 ± 0.85b	2.55 ± 0.58ab	2.94 ± 1.56ab	0.58 ± 0.21b	0.25 ± 0.11b
*Paraphelenchus*	0.03 ± 0.03	0.05 ± 0.04	0.06 ± 0.04	0.00 ± 0.00	0.00 ± 0.00	0.00 ± 0.00
**Herbivores**						
*Basiria*	2.23 ± 0.65ab	0.55 ± 0.21c	1.77 ± 0.44b	4.47 ± 1.62a	1.61 ± 0.45b	1.25 ± 0.48ab
*Boleodorus*	0.77 ± 0.21d	0.70 ± 0.18d	2.68 ± 0.76 cd	6.37 ± 1.46bc	10.89 ± 2.38a	8.05 ± 1.68ab
*Irantylenchus*	1.29 ± 0.28a	0.91 ± 0.25a	0.39 ± 0.08b	0.35 ± 0.11b	0.25 ± 0.06b	0.05 ± 0.03b
*Neopsilenchus*	3.62 ± 2.26a	0.37 ± 0.17b	0.07 ± 0.04b	1.41 ± 0.86ab	1.12 ± 0.34ab	0.65 ± 0.32b
*Pratylenchus*	7.07 ± 2.32b	1.39 ± 0.84c	2.26 ± 1.54c	42.59 ± 4.46a	13.14 ± 3.84b	7.83 ± 2.65bb
*Psilenchus*	0.15 ± 0.08	0.34 ± 0.10	0.35 ± 0.08	0.42 ± 0.14	0.18 ± 0.10	0.31 ± 0.13
**Predators**						
*Aporcella*	1.82 ± 1.24	0.31 ± 0.21	0.08 ± 0.08	3.93 ± 2.04	4.95 ± 2.40	5.67 ± 5.14
*Mesodorylaimus*	3.61 ± 2.28	2.36 ± 1.22	2.93 ± 1.17	0.13 ± 0.07	0.27 ± 0.17	1.50 ± 0.39
*Mylonchulus*	0.19 ± 0.06b	0.05 ± 0.02b	0.38 ± 0.14b	0.58 ± 0.38ab	0.133 ± 0.09b	2.81 ± 1.87a
*Oxydirus*	0.08 ± 0.05b	0.37 ± 0.18b	0.45 ± 0.17b	0.63 ± 0.25b	4.16 ± 2.23a	2.21 ± 0.51ab
*Prionchulus*	4.45 ± 0.98	1.89 ± 1.26	0.46 ± 0.33	0.09 ± 0.09	1.75 ± 1.46	0.00 ± 0.00
*Pristionchus*	0.04 ± 0.04b	9.71 ± 5.44a	0.12 ± 0.12b	0.00 ± 0.00b	1.63 ± 1.02b	0.01 ± 0.01b

The relative abundance of nematodes genera < 1% and identification at family level were excluded from the list. The values are mean ± standard error. Same letters are not statistically significant at *P <* 0.05 according to Tukey–Kramer test. CT = conventional tillage, MT = minimum tillage, NT = occasional tillage within no-tillage.

### Relative abundance of nematode feeding groups

**Fig. 3:**
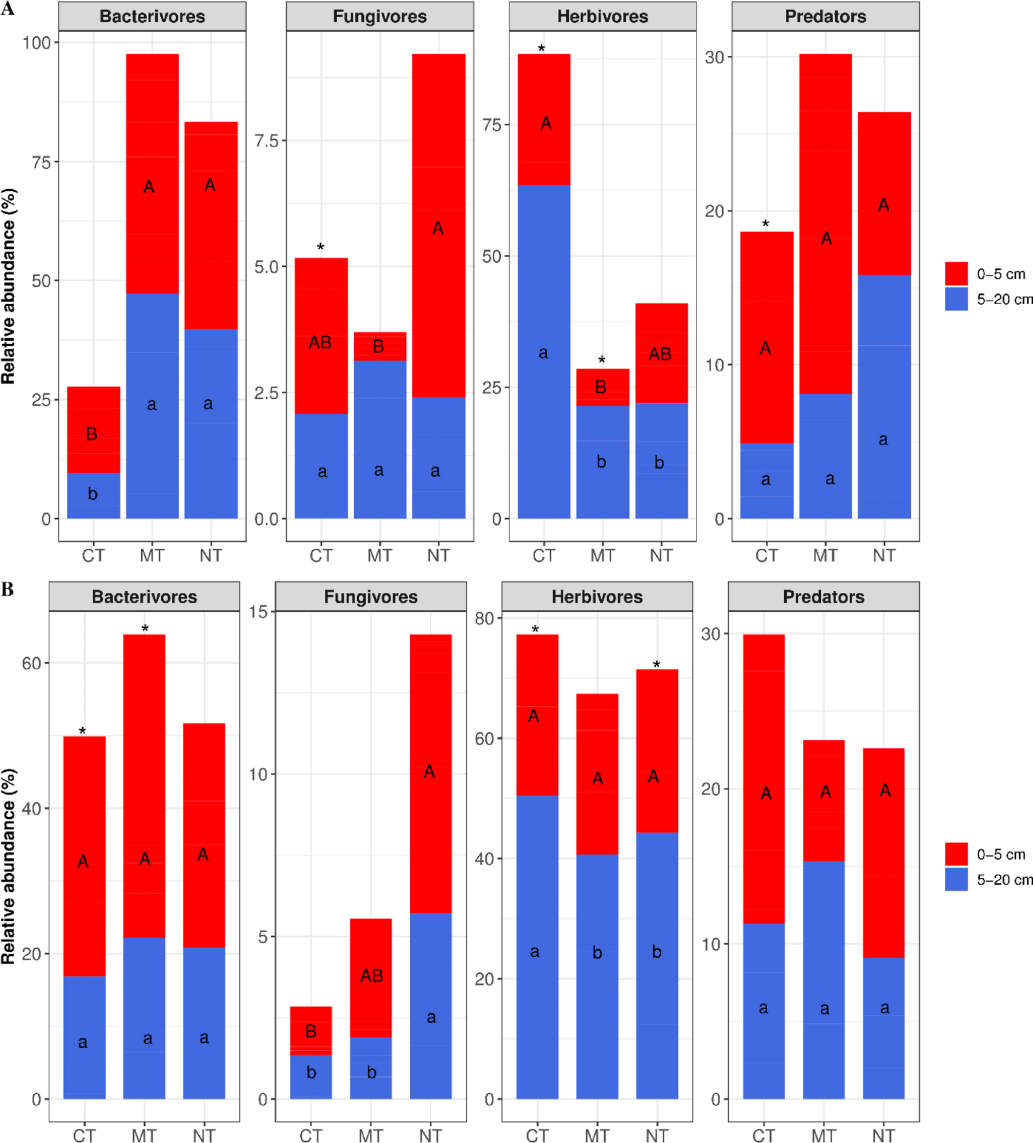
The effect of tillage on nematodes feeding groups in 2021 (**A**) and 2022 (**B**). Bars labeled with the same letter were not significantly different at *P* < 0.05. *Significant difference between sampling depth. CT = conventional tillage, MT = minimum tillage and NT = occassiona tillage within no–tillage. 0–5 cm depth, 5–20 cm depth.

**Fig. 4:**
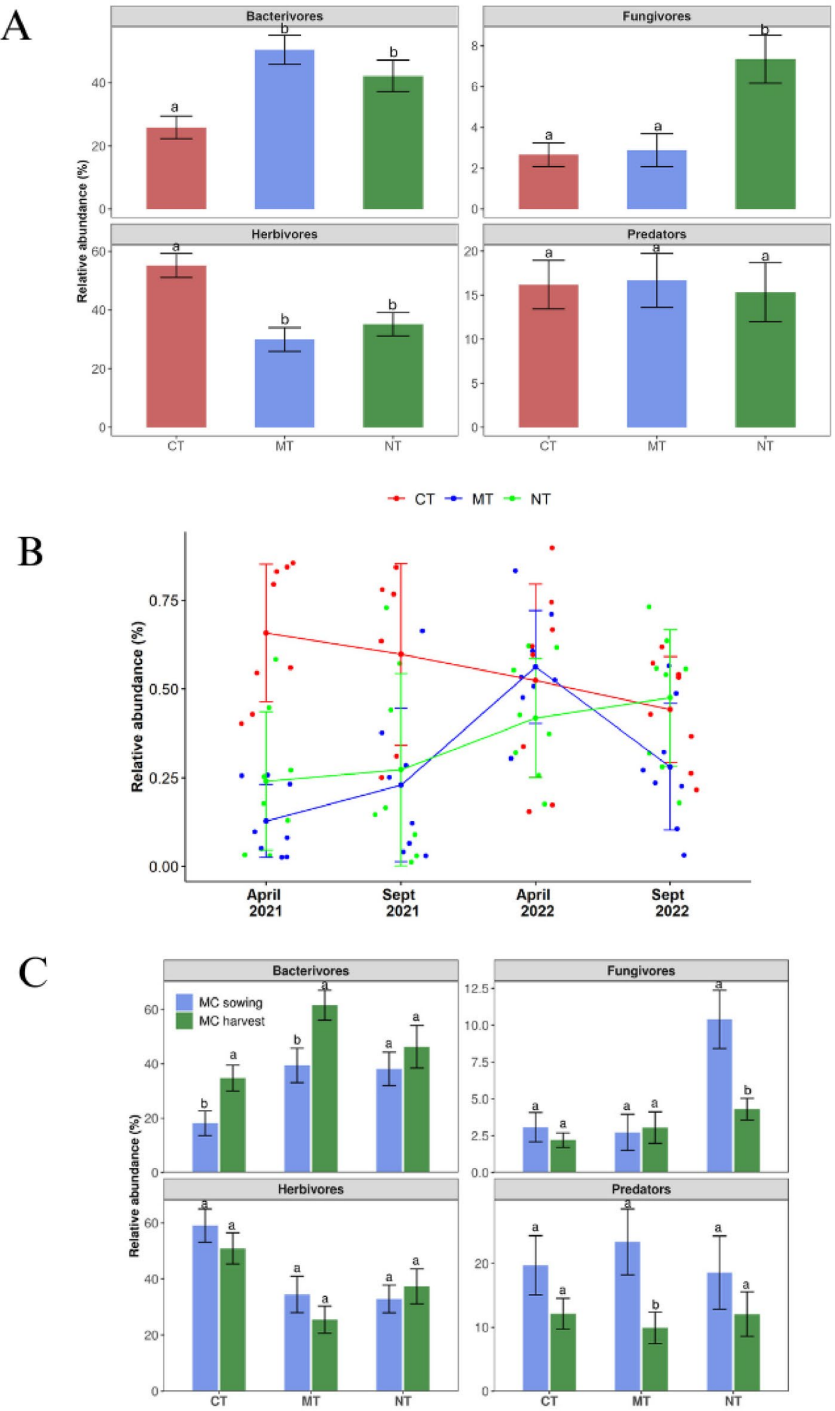
Soil nematode community strcture as affected by different tillage systems. Bar graph showing the relative abundance in different tillage systems (**A**), Variation in herbivore abundance at each sampling time (B), and Different nematode trophic groups at sowing and before harvesting of main crop (**C**). MC = main crop/cash crop.

The nematode community was predominantly composed of bacterivores and herbivores, regardless of sampling time or depth (Fig. 3), with each trophic group accounting for 38.8% of the total nematode community. Bacterivores were significantly more abundant (*P < 0.05*) in the MT and NT systems at the 5–20 cm depth in 2021 but not in 2022. The relative abundance was higher in all treatments at 0–5 cm (> 58%) (Fig. 3A). Fungivores were generally observed to be significantly more abundant in the NT systems compared to CT and MT in both 2021 and 2022 (Fig. 3A, B; *P* < 0.05). This pattern was consistent in both years although genus-level contributions varied (Table 1). Although the relative abundance of predator was the highest at 0–5 cm, there was no significant differences between tillage practices or sampling depths (*P > 0.05*) (Table 2; Fig. 3). Herbivores were more abundant (64%) at the 5–20 cm depth compared to 0–5 cm (36%). The CT supported higher relative abundance of herbivores in 2021, a pattern not observed in 2022. Overall, herbivores were significantly abundant in the CT, while the MT and NT favoured bacterivores (Fig. 4A). Over time, herbivore populations declined in CT but increased in NT (Fig. 4B). Also, the relative abundance of nematode assemblages differed at different stages of main crop growth (Fig. 4C). Bacterivore relative abundance was higher at main crop harvest than at the earlier stages. Fungivorous nematodes were significantly higher during the late stage of crop growth and relative abundance of herbivore increased toward later stages.

**Table 2:** Mixed effect model analyses testing the effects of tillage type (T), sampling depth (D), time of sampling (S) and T × S interactions on nematode feeding groups and nematode diversity indices.

Index	Tillage type (T)		Sampling depth (D)		Sampling time (S)		T x S	
	F–ratio	*P*–value	F–ratio	*P*–value	F–ratio	*P*–value	F–ratio	*P*–value
**Diversity indices**								
Richness	0.72	0.49	4.28	0.04	5.18	0.003	5.45	< 0.001
Shannon	0.91	0.41	5.7	0.02	12.59	< 0.0001	0.66	0.68
Simpson	0.86	0.43	3.58	0.06	10.07	< 0.0001	1.42	0.22
**Trophic abundance**								
BN	13.39	< 0.0001	15.2	< 0.001	6.32	0.01	3.24	0.001
HN	21.09	< 0.0001	42.03	< 0.0001	3.33	0.07	6.13	< 0.0001
FN	15.81	< 0.0001	3.7	0.06	2.85	0.1	4.96	< 0.001
PD	0.11	0.9	3.17	0.08	3.46	0.06	0.98	0.45
**Nematode indices**								
MI	11.31	< 0.0001	0.56	0.46	4.87	0.03	2.28	0.046
BI	6.99	0.002	3.06	0.08	6.91	0.01	1.07	0.39
CI	17.13	< 0.0001	3.78	0.06	3.33	0.07	0.7	0.65
NCR	7.13	0.002	0.55	0.46	4.99	0.003	4.39	0.001
EI	20.37	< 0.0001	4.85	0.03	14.28	< 0.001	3.3	0.007
SI	1.32	0.27	2.44	0.12	2.57	0.12	1.25	0.29
PPI	22.48	< 0.0001	47.64	< 0.0001	1.05	0.42	1.26	2.89
**Soil properties**								
pH	31.77	< 0.0001	21.35	< 0.0001	14.86	< 0.0001	5.84	< 0.0001
OM	11.59	< 0.0001	185.78	< 0.0001	2.73	0.049	1.31	0.27
CEC	16.80	< 0.0001	34.03	< 0.0001	6.01	0.001	8.96	< 0.0001
TN	2.65	0.078	22.33	< 0.0001	88.43	< 0.0001	2.09	0.065
TC	6.35	0.003	131.65	< 0.0001	1.21	< 0.0001	5.68	0.0001
TOC	13.89	< 0.0001	157.70	< 0.0001	3.88	0.01	2.05	0.07

BN = Bacterivore nematodes, HN = Herbivore nematodes, FN = Fungivore nematodes, PD = Predator nematodes. M I = Maturity index, BI = Basal Index, CI = Channel Index, NCR = Nematodes channel ratio, EI = Enrichment Index, SI = Structure index, PPI = Plant-parasitic index, OM = organic matter, CEC = cation exchange capacity, TN = total nitrogen, TC = total carbon, TOC = total organic carbon.

### Nematode community indices and food web structure

**Fig. 5:**
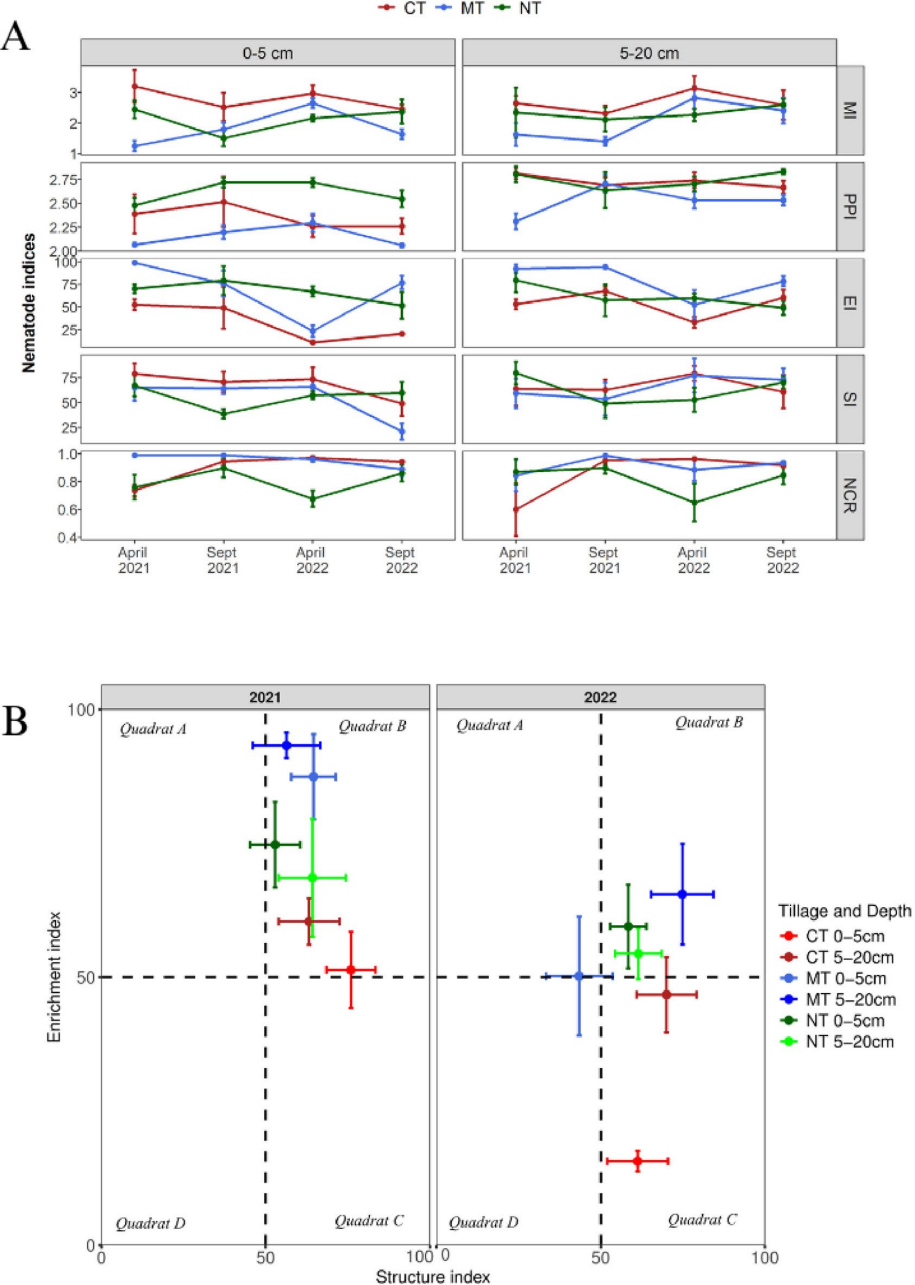
Soil food web analysis. Effect of tillage and depth of sampling on nematode community indices (**A**), Enrichment and structure indices for each tillage type and depth of sampling in 2021 and 2022 (**B**). PPI = plant-parasitic index, EI = enrichment index, SI = structure index, NCR = nematode channel ratio.

The effect of tillage on alpha diversity indices was not significant (*P* > 0.05) at either depth, but it did impact nematode community indices (Tables 2 and 3). The MI was significantly higher in CT but showed an increasing trend in NT (Table 3; Fig. 5A and Supplementary Fig. S1). The EI responded to tillage with greater values under MT and NT. The PPI was significantly higher under both CT and NT system. Although CT had higher SI values, it showed a decreasing trend whereas NT was increasing (Fig. 5A and S1). NCR values were higher in CT and MT, an indication of a progressive increase in bacterivore involvement in the decomposition pathway. The EI and SI, which reflect the maturity of the system, showed different food web conditions in 2021 and 2022 (Fig. 5B). In 2021, all tillage systems were in quadrant “B”, characterized by high nutrient availability, low disturbance, and decomposition channels dominated by bacterial and fungal pathways. In 2022, while NT remained in quadrant “B” at both depths, MT at 0–5 cm and 5–20 cm depths were in quadrants “A” and “B”, respectively. The CT system shifted to quadrant “C”, which was characterized by low nutrients, low disturbance, and decomposition dominated by fungal pathways.

**Fig. 6:**
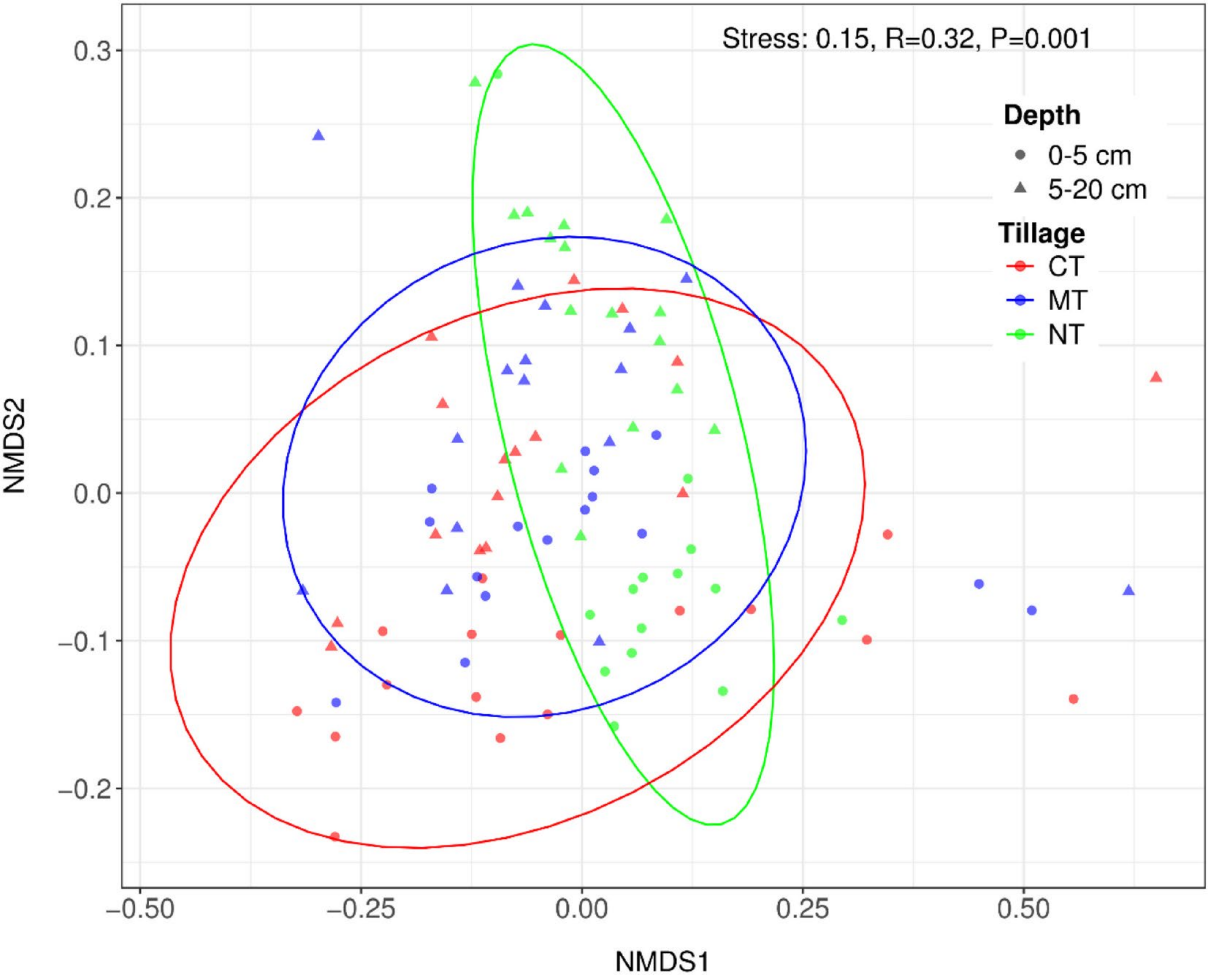
Non-metric multidimensional scaling (NMDS) of nematode community assessments under different tillage systems. Data points are distinguished by symbols for the depth of sampling and by color for the tillage types. The ellipses indicate 95% confidence intervals around group centroids.

The NMDS ordination showed that data points for all samples were separated according to sampling depth and tillage practices (Fig. 6). The ordination analysis revealed shifts in nematode community compositions among different tillage systems (*R* = 0.32, *P* = 0.001). PERMANOVA based on Bray–Curtis dissimilarity showed significant differences among the pairwise comparisons of tillage systems (Supplementary Table S3). The largest dissimilarity was observed between CT and NT (R² = 0.10, F = 6.83). To assess the assumption of homogeneity of multivariate dispersion, we performed a PERMDISP test and the results showed no significant differences in dispersion among treatments (F = 1.21, *P* = 0.31). This confirms that the observed differences in nematode community composition reflect genuine shifts.

**Table 3:** Nematodes ecological and alpha diversity indices in different tillage practices.

Index	0–5 cm			5–20 cm		
	CT	MT	NT	CT	MT	NT
Shannon	2.20 ± 0.10a	1.83 ± 0.14a	2.06 ± 0.14a	1.88 ± 0.10a	1.90 ± 0.14a	1.86 ± 0.12a
Richness	22.00 ± 1.35a	22.90 ± 1.91a	23.90 ± 1.41a	20.20 ± 1.26a	21.50 ± 1.33a	20.90 ± 1.51a
Simpson	0.83 ± 0.02a	0.71 ± 0.05a	0.75 ± 0.05a	0.73 ± 0.04a	0.72 ± 0.05a	0.72 ± 0.04a
MI	2.82 ± 0.19a	1.83 ± 0.16b	2.12 ± 0.16b	2.67 ± 0.18a	2.06 ± 0.21a	2.32 ± 0.22a
BI	26.69 ± 5.23a	17.04 ± 3.96a	19.03 ± 3.25a	19.67 ± 2.84a	9.16 ± 2.88b	17.92 ± 2.71ab
EI	30.96 ± 5.79b	68.82 ± 8.14a	67.10 ± 5.73a	53.57 ± 4.37b	79.38 ± 5.90a	61.49 ± 6.07ab
PPI	2.33 ± 0.07b	2.15 ± 0.04c	2.61 ± 0.04a	2.73 ± 0.04a	2.52 ± 0.05b	2.74 ± 0.05a
CI	55.73 ± 6.99a	6.73 ± 2.66c	27.51 ± 6.86b	23.47 ± 7.18a	7.73 ± 2.99b	26.00 ± 6.94a
NCR	0.89 ± 0.03ab	0.96 ± 0.02a	0.80 ± 0.04b	0.86 ± 0.06a	0.91 ± 0.03a	0.81 ± 0.05a
SI	67.55 ± 6.29a	54.01 ± 6.49a	55.64 ± 4.62a	66.63 ± 6.33a	65.61 ± 7.15a	62.85 ± 6.04a

The values are mean ± standard error (*n* = 4). Same letters are not statistically significant at *P* < 0.05 according to Tukey–Kramer test. Pairwise comparison of treatment was performed at each sampling depth. MI = Maturity index, BI = basal index, EI = enrichment index, PPI = plant-parasitic index, CI = channel index, NCR = nematode channel ratio, SI = structure index.

### Relationship between soil properties and nematode community

**Fig. 7:**
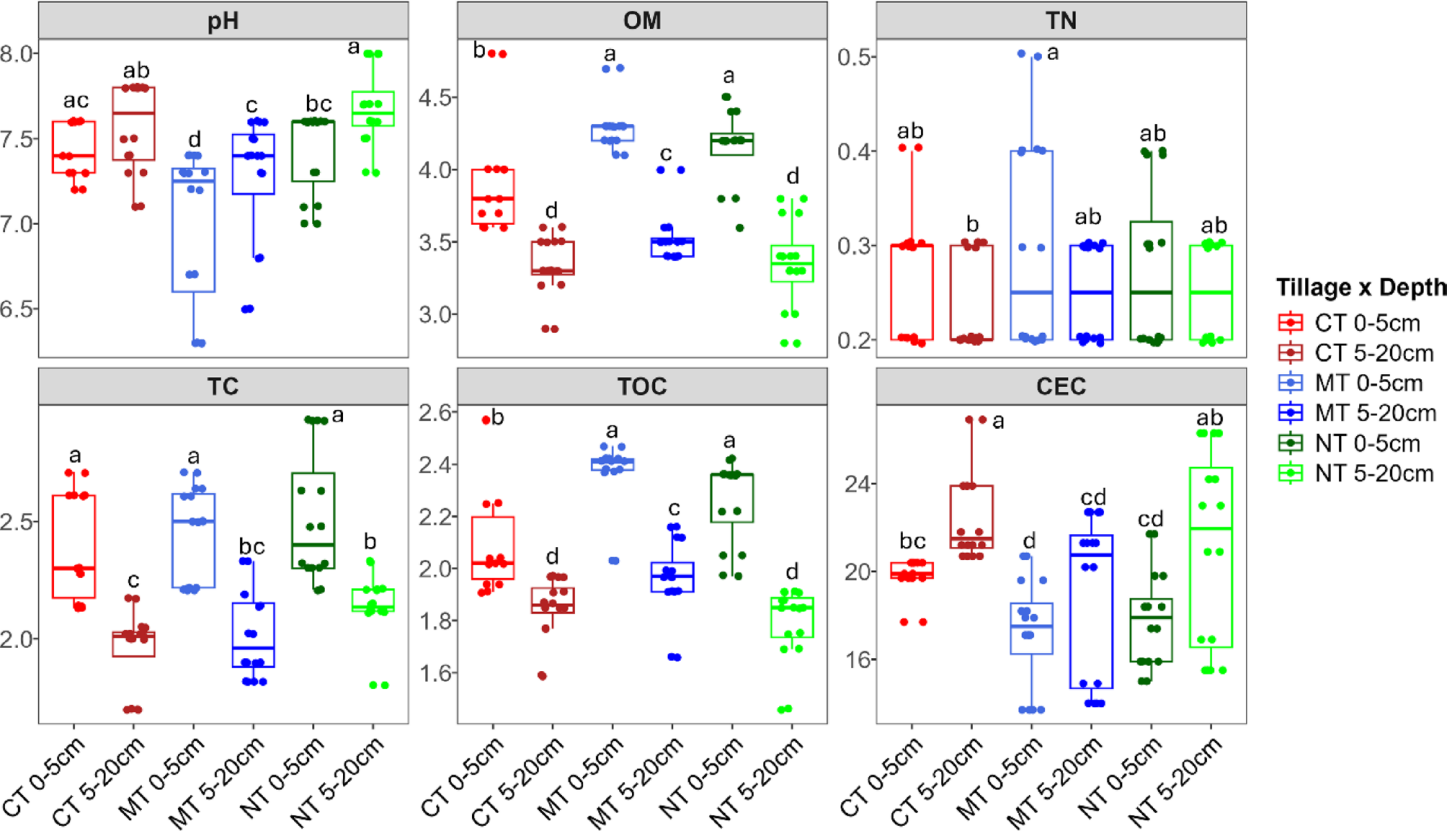
Soil properties in different tillage systems and depths of sampling. Bars labeled with the same letter are not significantly different at *P* < 0.05 according to Tukey–Kramer test. OM = organic matter, CEC = cation exchange capacity, TC = total carbon, TN = total nitrogen, TOC = total organic carbon.

Soil OM, TC and TOC were significantly higher (*P* < 0.005) at 0–5 cm compared to 5–20 cm depth (Fig. 7). On the contrary, soil pH and CEC were lowest at 0–5 cm and highest at 5–20 cm depth. Specifically, soil pH at 5–20 cm in the NT system was significantly higher (*P* < 0.05) than at the 0–5 cm depth. Similarly, the CEC was significantly higher at 5–20 cm in the CT and NT than at the 0–5 cm depth. There was no significant difference in TN at different depths of sampling for all tillage systems (Fig. 7).

**Fig. 8:**
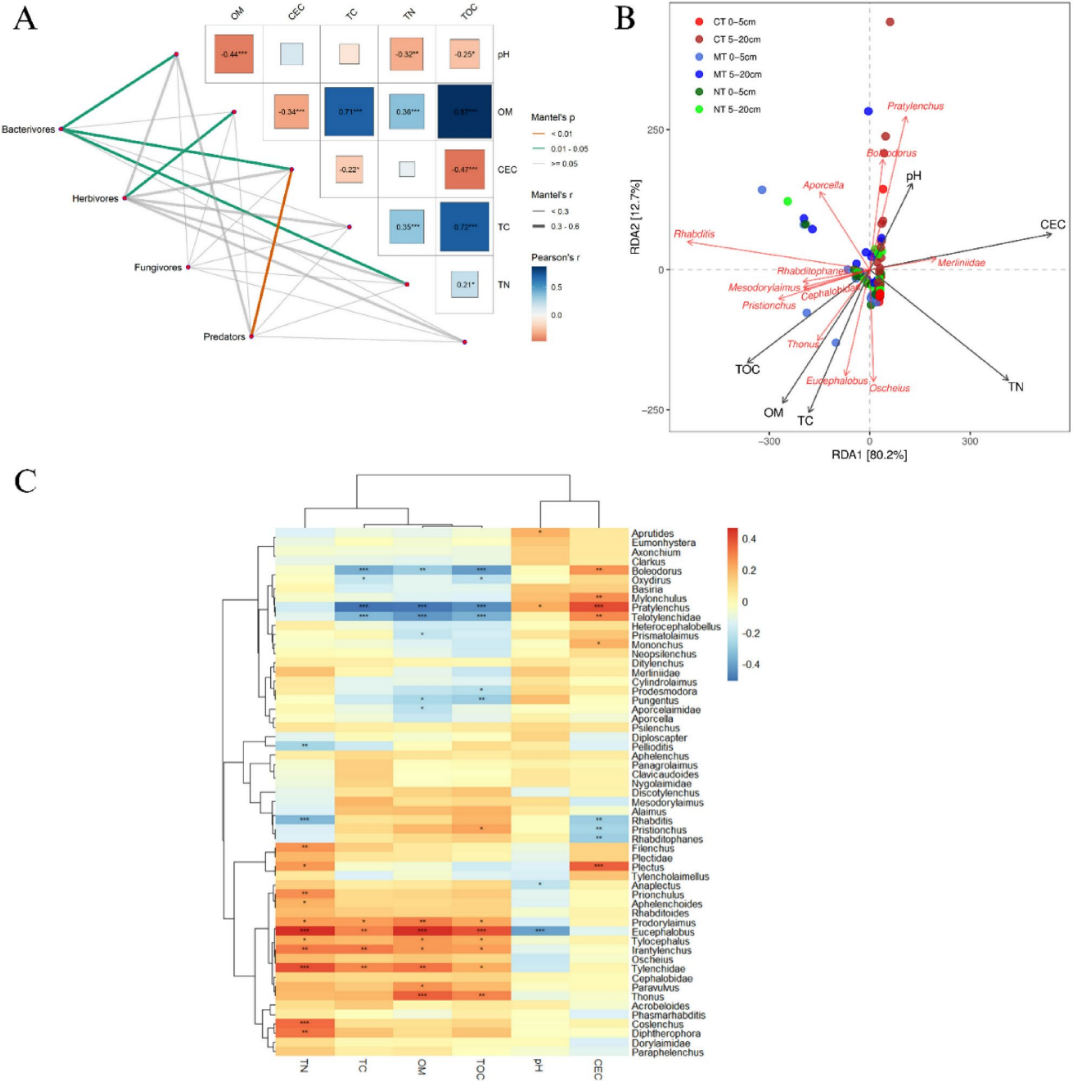
Partial mantel correlation test (**A**), redundancy analyses (**B**), and heatmap (**C**) based on soil properties and nematode communities. The partial Mantel’s r statistic is represented by line width, and the colour of the line indicates the statistical significance. Asterisk are Pearson’s correlations based on pairwise comparisons of soil properties (*, *P* < 0.05; **, *P* < 0.01; ***, *P* < 0.001).

 The abundance of nematode communities was also found to be influenced by soil characteristics in varying ways (Table 2). The partial Mantel test analysis showed that soil properties were significantly related to the nematode communities (Fig. 8A). pH, CEC and TN (Mantel’s *r* > 0.3, Mantel’s *P* < 0.05) were correlated with the bacterivores. CEC was found to be the dominant factor in determining the predator feeding group. There was no correlation between fungivores and soil properties (Fig. 8A). Moreover, the results revealed that OM was strongly correlated with herbivore community structure (Mantel’s *r* > 0.3, Mantel’s *P* < 0.05).

The RDA results corroborated the variations in soil nematode community composition, soil characteristics, and the various tillage techniques discussed earlier (Fig. 8B). The first two axes of RDA explained 80.2% and 12.7% of the total variation in the nematode community. The genera *Pratylenchus* and *Boleodorus* were positively correlated with soil pH and CEC, and negatively associated with TC, TOC and OM (Fig. 8A and C). Beneficial free–living nematode genera, viz., *Mesodorylaimus*,* Pristionchus*, *Thonus Cephalobidae*, *Oscheius* and *Rhabditis* were positively associated with increased TOC, OM, and TC in the MT and NT systems.

## Discussion

No–tillage is increasingly recognized as a sustainable agricultural production system^15,18^. However, growing concerns of the negative impact of no–tillage especially in clayey and wet soils have generated interest in occasional tillage within no–tillage (NT) and in understanding how this influences soil ecosystems^23^. As studies have demonstrated the benefits of NT on the soil, crops and environment^22,46^it is important to gain a better understanding of its implications for soil microbial communities including nematodes. Therefore, to assess the sustainability of NT practices in Canada’s corn–soybean production, their impact on nematode communities must be understood.

Depending on the level of disturbance exerted on the soil, it can influence the nematode communities and their hierarchical distribution within the soil profile^21^. In our experiment, the beneficial free–living nematodes (bacterivores, fungivores, and predators) dominated the upper part of the soil (0–5 cm), while the 5–20 cm depth was inhabited by herbivores. The higher abundance of free-living nematode is attributed to the concentration of microbial biomass in the topsoil^21,25^. Bacterivores relative abundance was significantly higher in the MT and NT system at both depths in 2021 but this pattern did not persist in 2022. This temporal variability was explained by the findings of Zhong, et al.^47^who stated that tillage effects can vary annually depending on climatic conditions and crop functionality. Additional reports have documented a decrease in bacterivore under CT^21,47^. Zhong, et al.^47^ attributed the lower abundance of bacterivores in the CT to damage by the tillage equipment or desiccation. Our results could not confirm this reasoning as there was also a reduction in the population of bacterivores at the 5–20 cm depth. The higher relative abundance of bacterivores in MT and NT significantly increased *Rhabditis* abundance. This genus is known for being a resource enrichment opportunist, thriving in systems with high crop resource availability^48^. However, some studies have reported higher bacterivore abundance in CT than in reduced or no-tillage^24,49^. Bacterivore populations peaked at crop maturity in the MT and CT, with more than a 1.7 fold increase in MT relative to CT. Findings by Qiao, et al.^50^ confirm our results, as they also observed higher abundance of bacterivore under CT and MT at harvest time. This suggests bacterivores respond positively to plant resources, which are available later in the growing season. Fungivore relative abundance was generally low across tillage practices, although NT supported a higher proportion of fungivores, especially at the 0–5 cm depth. The absence of physical disturbance has been shown to favour fungal growth especially at the 0–5 cm depth resulting in positive bottom-up effect on fungivores^11,18^. The increased abundance of bacterivores and fungivores in NT suggests that this system can sustain both groups. Contrary to our second hypothesis, there was no significant difference in the predator populations across the different tillage practices and depth of soil sampling. However, they were prevalent at the 0–5 cm depth range and according to Sánchez-Moreno and Ferris^51 ^this may enhance the soil’s capacity to control pest outbreaks. It was anticipated that MT and NT would help augment predatory nematodes, which are sensitive to disturbances^47,52,53^. Our results are an indication that the five–year period was not long enough to attain the effect of MT and NT on predator nematodes due to their long generation times^6,51^.

In agreement with past studies, tillage practices have a variable influence on herbivores^21,47,54^. Unlike free–living nematodes, a higher prevalence of herbivores such as *Boleodorus* and *Pratylenchus* was observed at 5–20 cm than at the 0–5 cm depth. These differences could be explained by the location of the corn/soybean root systems that extend beyond the 0–5 cm depth. Although these genera differ in their mode of parasitism, their association with deeper root zones may explain their higher relative abundance at the 5–20 cm depth^55^. The relative abundance of herbivore was significantly higher (> 42%) in CT than in reduced tillage systems at both depths. Well-documented evidence shows that CT disrupts soil structure and natural ecological checks^22,56 ^leading to favourable conditions for herbivores to thrive. This could also explain the lack of significant differences between tillage types in 2022, when tillage was carried out in all treatments. Our results are supported by Sánchez-Moreno and Ferris^57^ and Zhang et al.^53^ who demonstrated that tillage reduced food web complexity and favoured herbivore dominance.

The CT system was dominated by the root lesion nematode *Pratylenchus*, which accounted for 47% of herbivores at 0–5 cm and 76% at 5–20 cm. *Pratylenchus* nematodes are the most common and economically damaging pests in Canadian crop production^58^. These nematodes are widespread and was found in 42% of the tested fields in the Province of Quebec^59^. Therefore, avoiding practices that may inadvertently promote its increase is crucial to prevent crop yield losses. Furthermore, we observed a worrying trend of increasing relative abundance of herbivore such as *Pratylenchus* in the NT system, whereas the population was declining under CT. The increasing prevalence of the nematode in the NT in our study can be related to the reduction in tillage and potential association with weed hosts. However, further research is needed to clarify the role of weed reservoirs, especially given the wide host range of this genus. Another important herbivorous nematodes identified at the study site was Merliniidae spp., which are commonly associated with grain crops^60,61^. Interestingly, Merliniidae were consistently abundant in the NT system in both years and all sampling phases. Conversely, CT and MT had the greatest impact on Merliniidae abundance, decreasing their numbers across the sampling years. Similar trends have been reported in previous studies, suggesting that soil disturbance associated with tillage negatively impacts the persistence of these nematode genera^61,62^. The contrasting trends in herbivore populations across tillage systems may have important implications for long-term soil sustainability and pest management. Under CT, the reduced relative abundance of herbivoreparticularly *Pratylenchus*–may be associated with repeated physical disturbance, which impairs nematode mobility and survival^26^. In contrast, the gradual increase in herbivores under NT may indicate that reduced soil disturbance creates a more stable environment, allowing certain herbivore genera to persist^63^. Furthermore, the observed increase in herbivorous nematode populations under NT depicts the need for proactive IPM strategies. NT systems foster soil structure and moisture which benefits overall soil health by creating conditions conducive to nematode persistence, particularly for herbivores prone to damaging corn and soybean roots^64^. To maintain long-term pest control under NT, growers should consider strategies such as crop rotation, resistant varieties, and targeted cover cropping to disrupt nematode life cycles and suppress pest buildup^65^.

Changes in nematode community structure due to tillage were reflected in the nematode’s ecological indices^47,54^. The CT system which exerts the greatest soil disturbance can, in some cases, improve soil micro-ecological stability with increasing MI^50^. In agreement with our experiment, the CT had exhibited the highest MI especially at the 0–5 cm depth. The elevated MI and SI observed under CT could be explained by the possibility that repeated disruption requires an extended period before it significantly alters soil food web structure and stability^50^. Interestingly, the increasing trend of MI and SI in NT and MT could be linked to the susceptibility of K–selected nematodes to disturbance but they can recover when tillage intensity is reduced^51^. The relatively higher EI in MT and NT systems across the soil depths suggests increased nutrient availability from bacterivore activity^66^. However, the lower NCR observed in NT indicates a gradual transition in the soil decomposition pathway from bacterial–mediated to fungal–mediated. The shift indicates a progressive adaptation of the soil community to undisturbed conditions, fostering fungal growth^49^. This observation on nematode community shift was also reported previously^67^. In the long term, this alteration may lead to slower nitrogen cycling and decomposition rates, as a fungal–dominated system generally results in reduced nutrient turnover^68^. Based on our results, this community shift in decomposition pathways is not expected to significantly affect overall nutrient composition, as NT systems have shown to promote optimal soil health conditions. The PPI was higher in CT and NT systems, but the composition of plant-parasitic nematodes differed among treatment groups. CT and MT favoured nematode genera within Pratylenchidae family, while NT supported a higher abundance of Merliniidae. This could indicate a shift in herbivore composition, reflecting differential tolerance to tillage regimes and environmental filtering.

The present study further investigated the interaction between tillage practices and various soil physicochemical properties and their influence on nematode community dynamics. Tillage systems had a significant impact on the depth distribution of soil properties. In the NT and MT systems, the OM and TOC were markedly higher at the 0–5 cm depth. This is in agreement with studies from Paye, et al.^69^ and Peixoto, et al.^22^who reported that surface accumulation of carbon is associated with MT and NT due to limited mixing of crop residues. Soil pH and CEC were lowest in the 0–5 cm depth and higher in the 5–20 cm depth in the NT and MT systems. Peixoto, et al.^22^ suggested NT as a strategic practice to mitigate this depth-dependent nutrification variation. These findings confirm that while NT reduces mechanical soil disturbance, it may also lead to depth-dependent nutrient stratification. Long-term nutrient availability has practical implications for soil health management as nutrient stratification may limit root access and alter the microbial communities. The RDA analysis indicates that the improved soil properties of the NT and MT systems positively influenced free–living nematodes. Furthermore, the Mantel test revealed that, pH, CEC and TN were key drivers of nematode community structure, aligning with findings from broader regional studies^70^. Moreover, the differential responses among the nematode feeding groups suggest that their distinct ecological roles and adaptive strategies contribute to varying reactions to environmental changes^9,11^.

## Conclusion

The results of the research indicate that tillage methods influence nematode group richness, composition, and trophic structure. MT and NT can be promising agricultural management in clayey soils, promoting the relative abundance of bacterivorous nematodes and EI values, an indication of improved nutrient cycling potential. The CT can initially increase herbivore abundance but their populations appeared to decline over time, likely due to reduced habitat stability. Surprisingly, the NT system showed a gradual increase in herbivore populations. However, the system might be self-regulating, as an increased SI over time is expected to enhance soil suppressiveness and promote bottom-up control of soil-borne pests, including herbivores. Although, MT and NT improve the nutrient content in surface layers, vertical nutrient stratification remains a concern. Future research should focus on determining the optimal depth and frequency of occasional tillage to balance nutrient redistribution while preserving soil health benefits. Overall, integrating nematode ecological indices with soil properties provides key insights into the mechanistic pathways by which reduced and occasional tillage influence soil food web dynamics. The integrated approach offers a more holistic framework for evaluating sustainable soil tillage practices in agriculture.

## Electronic supplementary material

Below is the link to the electronic supplementary material.

Supplementary Material 1

## Data Availability

The raw sequence generated and analysed during the current study are available in the GenBank Sequence Read Archive (SRA) under accession number PRJNA1253153.
