# Supplementary material for: Metabarcoding–approach–based profiling reveals dynamic nature of sustainable tillage practices on nematode communities in corn–soybean cropping systems
Source: Sci Rep. 2025 Jul 14;15:25372. doi: 10.1038/s41598-025-09356-6 (PMC12260074; doi:10.1038/s41598-025-09356-6)
Supplement: Supplementary file 1 — Supplementary Material 1 [file 41598_2025_9356_MOESM1_ESM.docx]

**Table S1.** Historical perspective of field activities performed from 2018 to 2022 for the conventional tillage (CT), minimum tillage (MT), and occasional tillage within no-tillage (NT).

| **System** | **Year** | **Crop type** | **Tillage** | **Depth (cm)** | **Manure** |
| --- | --- | --- | --- | --- | --- |
| CT | 2018 | Corn | Mouldboard plough, Kongskilde cultivator and high–speed disc pass | 25 | LM (cattle, hog and poultry) – 7.4 Mgha^-1^ |
|  | 2019 | Soybean | Mouldboard plough, Kongskilde cultivator and high–speed disc pass. | 25 | LM (cattle, hog and poultry) – 7.4 Mgha^-1^ |
|  | 2020 | Corn | Mouldboard plough, Kongskilde cultivator and high–speed disc pass | 25 | LM (cattle, hog and poultry) – 7.4 Mgha^-1^ |
|  | 2021 | Soybean | Mouldboard plough, Kongskilde cultivator and high–speed disc pass | 25 | LM (cattle, hog and poultry) – 7.4 Mgha^-1^ |
|  | 2022 | Corn | Mouldboard plough, Kongskilde cultivator and high–speed disc pass | 25 | LM (cattle, hog and poultry) – 7.4 Mgha^-1^ |
| MT | 2018 | Corn | Kongskilde cultivator and high–speed disc pass | 15 | LM (cattle, hog and poultry) – 7.4 Mgha^-1^ |
|  | 2019 | Soybean | Kongskilde cultivator and high–speed disc pass | 15 | LM (cattle, hog and poultry) – 7.4 Mgha^-1^ |
|  | 2020 | Corn | Kongskilde cultivator and high–speed disc pass | 15 | LM (cattle, hog and poultry) – 7.4 Mgha^-1^ |
|  | 2021 | Soybean | Kongskilde cultivator and high–speed disc pass | 15 | LM (cattle, hog and poultry) – 7.4 Mgha^-1^ |
|  | 2022 | Corn | Kongskilde cultivator and high–speed disc pass | 15 | LM (cattle, hog and poultry) – 7.4 Mgha^-1^ |
| NT | 2018 | Corn | - | - | LM (cattle, hog and poultry) – 7.4 Mgha^-1^ |
|  | 2019 | Soybean | High–speed disc pass | 15 | LM (cattle, hog and poultry) – 7.4 Mgha^-1^ |
|  | 2020 | Corn | - | - | LM (cattle, hog and poultry) – 7.4 Mgha^-1^ |
|  | 2021 | Soybean | - | - | LM (cattle, hog and poultry) – 7.4 Mgha^-1^ |
|  | 2022 | Corn | High–speed disc pass | 15 | LM (cattle, hog and poultry) – 7.4 Mgha^-1^ |

LM = Liquid manure.

**Table S2.** Analysis of soil properties and the procedures followed for their measurements.

| **Variable (units)** | **Abbreviation** | **Method^y^** |
| --- | --- | --- |
| Cation exchange capacity (meq/100g of soil) | CEC | Ammonium displacement |
| Total nitrogen (%) | TN | Combustion, TMECC |
| Total organic carbon | TOC | Combustion, TMECC |
| Total carbon (%) | TC | Combustion, TMECC |
| Organic matter (LOI%) | OM | Loss on ignition |
| pH | pH | 1:1 Soil: Water |
| Sand (%) | - | MSA Part 1 (1986) pp 404-408 |
| Silt (%) | - | MSA Part 1 (1986) pp 404-408 |
| Clay (%) | - | MSA Part 1 (1986) pp 404-408 |

^y^Methods from Jones^27^ and Gardner ^28^.

**Table S3**. PERMANOVA to assess the impact and significance of tillage and depth of soil sampling on nematode communities.

| **Parameter** | **R^2^** | **F** | **Pr(>F)** |
| --- | --- | --- | --- |
| MT vs NT | 0.04 | 2.86 | 0.003 |
| MT vs CT | 0.09 | 6.43 | 0.001 |
| NT vs CT | 0.10 | 6.83 | 0.002 |
| MT 0-5cm vs 5-20 cm | 0.09 | 3.32 | 0.001 |
| NT 0-5cm vs 5-20 cm | 0.07 | 2.22 | 0.023 |
| CT 0-5cm vs 5-20 cm | 0.20 | 6.96 | 0.001 |
| 0-5 cm MT vs NT | 0.07 | 2.20 | 0.041 |
| 0-5 cm MT vs CT | 0.12 | 3.76 | 0.001 |
| 0-5 cm NT vs CT | 0.13 | 4.14 | 0.001 |
| 5-20 cm MT vs NT | 0.06 | 1.95 | 0.027 |
| 5-20 cm MT vs CT | 0.14 | 5.08 | 0.001 |
| 5-20 cm NT vs CT | 0.16 | 5.69 | 0.001 |


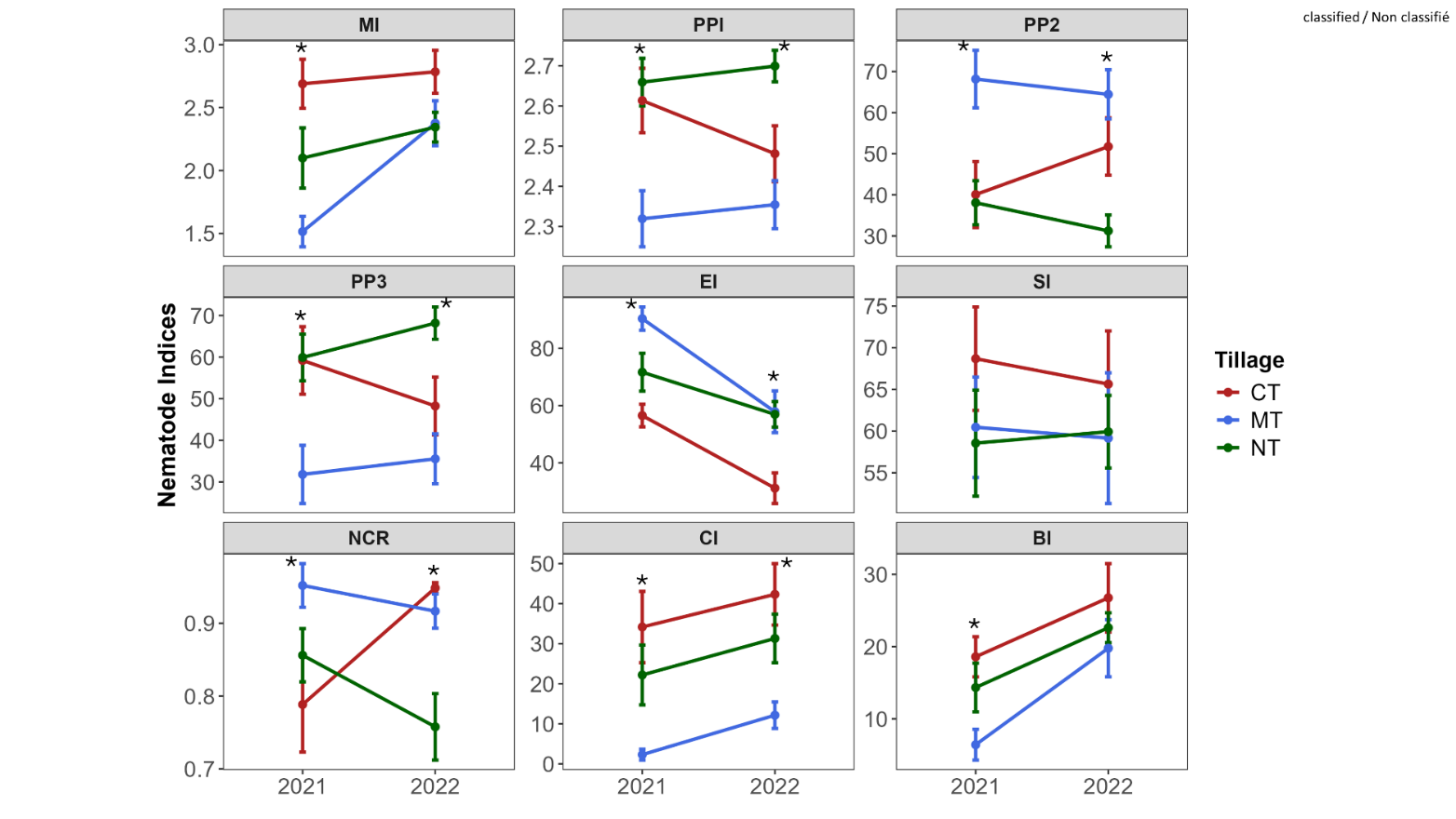


**Fig. S1:** Effect of tillage on nematode community indices. Data were combined across the sampling regardless of the depth (n=16). Asterisk * on each line show significant differences at p < 0.05 (Tukey’s HSD) within each year. CT = conventional tillage, MT = minimum tillage and NT = occasional tillage within no-tillage.
